# Variation in antibiotic consumption in very preterm infants—a 10 year population-based study

**DOI:** 10.1093/jac/dkad358

**Published:** 2023-11-21

**Authors:** Zuzana Huncikova, Hans Jørgen Stensvold, Knut Asbjørn Alexander Øymar, Anlaug Vatne, Astri Maria Lang, Ragnhild Støen, Anne Karin Brigtsen, Dag Moster, Beate Horsberg Eriksen, Terje Selberg, Arild Rønnestad, Claus Klingenberg

**Affiliations:** Paediatric Department, Stavanger University Hospital, Stavanger, Norway; Department of Clinical Science, University of Bergen, Bergen, Norway; Department of Neonatal Intensive Care, Clinic of Paediatric and Adolescent Medicine, Oslo University Hospital, Oslo, Norway; Paediatric Department, Stavanger University Hospital, Stavanger, Norway; Department of Clinical Science, University of Bergen, Bergen, Norway; Paediatric Department, Stavanger University Hospital, Stavanger, Norway; Paediatric Department, Akershus University Hospital, Lørenskog, Norway; Department of Paediatrics, St.Olavs Hospital, Trondheim University Hospital, Trondheim, Norway; Department of Clinical and Molecular Medicine, Norwegian University of Science and Technology, Trondheim, Norway; Department of Neonatal Intensive Care, Clinic of Paediatric and Adolescent Medicine, Oslo University Hospital, Oslo, Norway; Department of Paediatrics and Adolescent Medicine, Haukeland University Hospital, Bergen, Norway; Department of Global Public Health and Primary Care, University of Bergen, Bergen, Norway; Department of Paediatrics, Møre and Romsdal Hospital Trust, Ålesund, Norway; Clinical Research Unit, Norwegian University of Science and Technology, Trondheim, Norway; Department of Paediatric and Adolescent Medicine, Ostfold County Hospital, Gralum, Norway; Department of Neonatal Intensive Care, Clinic of Paediatric and Adolescent Medicine, Oslo University Hospital, Oslo, Norway; Department of Paediatric and Adolescent Medicine, Ostfold County Hospital, Gralum, Norway; Medical Faculty, Institute for Clinical Medicine, University of Oslo, Oslo, Norway; Department of Paediatrics and Adolescent Medicine, University Hospital of North Norway, Tromsø, Norway; Paediatric Research Group, Faculty of Health Sciences, UiT-The Arctic University of Norway, Tromsø, Norway

## Abstract

**Objectives:**

Wide variations in antibiotic use in very preterm infants have been reported across centres despite similar rates of infection. We describe 10 year trends in use of antibiotics and regional variations among very preterm infants in Norway.

**Patients and Methods:**

All live-born very preterm infants (<32 weeks gestation) admitted to any neonatal unit in Norway during 2009–18 were included. Main outcomes were antibiotic consumption expressed as days of antibiotic therapy (DOT) per 1000 patient days (PD), regional variations in use across four health regions, rates of sepsis and sepsis-attributable mortality and trends of antibiotic use during the study period.

**Results:**

We included 5296 infants: 3646 (69%) were born at 28–31 weeks and 1650 (31%) were born before 28 weeks gestation with similar background characteristics across the four health regions. Overall, 80% of the very preterm infants received antibiotic therapy. The most commonly prescribed antibiotics were the combination of narrow-spectrum β-lactams and aminoglycosides, but between 2009 and 2018 we observed a marked reduction in their use from 100 to 40 DOT per 1000 PD (*P* < 0.001). In contrast, consumption of broad-spectrum β-lactams remained unchanged (*P* = 0.308). There were large variations in consumption of vancomycin, broad-spectrum β-lactams and first-generation cephalosporins, but no differences in sepsis-attributable mortality across regions.

**Conclusions:**

The overall antibiotic consumption was reduced during the study period. Marked regional variations remained in consumption of broad-spectrum β-lactams and vancomycin, without association to sepsis-attributable mortality. Our results highlight the need for antibiotic stewardship strategies to reduce consumption of antibiotics that may enhance antibiotic resistance development.

## Introduction

Antibiotics are globally the most commonly prescribed medications in neonatal ICUs (NICUs).^1–4^ The majority of very preterm infants receive antibiotics and often prolonged courses.^1,5–7^ However, despite often similar rates of infections and microbial resistance profiles, there are wide variations in empirical antibiotic use across NICUs, both within and between countries.^3,5,8–10^ It is a valid question to ask why this occurs. In other parts of paediatric medicine, exemplified by paediatric oncology, strict treatment protocols are followed across national and international centres.^11^ Antimicrobial stewardship programmes (ASPs) advise clinicians on optimal empirical antibiotic therapy. Lack of high-quality clinical trial data^12,13^ combined with limited pharmacokinetic/pharmacodynamic data on antibiotics in neonates creates challenges when clinical experts try to agree on treatment guidelines.

Over the last decades it has been increasingly acknowledged that antibiotic overuse among very preterm infants is not only associated with increasing antibiotic resistance^14^, but also with severe adverse effects such as increased risk of invasive candidiasis, necrotizing enterocolitis (NEC), bronchopulmonary dysplasia (BPD) and death.^4,5,7,15–17^ Adequate choice of antibiotic regimens and the treatment duration, both for empirical and targeted therapy, are therefore important elements in order to reduce these adverse effects. Empirical therapy should ideally be based on local microbial resistance profiles, and also limit exposure to broad-spectrum antibiotics, minimizing potential adverse neonatal outcomes and emergence of MDR organisms.^6,7,10,16^ Some antibiotic classes are also associated with higher rates of nephrotoxicity, which may further compromise care of vulnerable preterm infants.^18,19^

Surveillance of antibiotic consumption is an important element of any ASP.^20^ Moreover, a factual approach quantifying the burden of treatment in relation to the burden of disease may contribute to optimize the delicate balance between antimicrobial stewardship and effective sepsis management.^21^ In the absence of data from clinical trials, high-quality, population-based studies are good alternatives to elucidate these complex topics. The primary aim of this study was to describe total antibiotic consumption, trends over time and regional variations among very preterm infants in Norway. Secondly, we aimed to assess whether there were associations between antibiotic consumption and regional variations in rates of neonatal sepsis and sepsis-attributable mortality.

## Methods

### Setting, data source and ethics

In 2021, Norway had a population of around 5.4 million people. Public healthcare is free of charge for all inhabitants. Hospital services are organized in four health regions covering populations of different sizes and both urban and rural areas. In the South-East region (*n* ∼ 3.1 million) the Oslo capital region has three large NICUs, in the West region (*n* ∼ 1.1 million) Stavanger and Bergen have the two largest NICUs, in the Mid region (*n* ∼ 0.75 million) Trondheim and Ålesund have the two largest NICUs and in the North Region (*n* ∼ 0.48 million) Bodø and Tromsø have the two largest NICUs. Socioeconomic status in Norway is fairly equally distributed, but the proportion of immigrants is highest in the South-East region.

Data for this study were obtained from the Norwegian Neonatal Network (NNN), a national population-based registry collecting data from all 20 Norwegian NICUs covering all four regional health regions. The registry includes daily registrations of investigations, treatments and diagnoses for all infants admitted to Norwegian NICUs.^22,23^ The NNN collects personal identifiable data without the need for consent, according to regulations for the Medical Birth Registry of Norway and the Norwegian Personal Health Data Filling System Act. The Regional Ethical Committee for medical and health research ethics approved this study (REK Helse Sør Øst 2012/944-1).

### Study population, data and definitions

This study includes all live-born preterm infants below 32 weeks gestation admitted to NICUs in Norway during the 10 year period from 1 January 2009 to 31 December 2018. We retrieved background data including year of birth, mode of delivery, birth weight (BW), gestational age (GA), clinical risk index for babies 2 (CRIB2) score,^24^ Apgar score, sex, antenatal steroids and clinical data including any antibiotic use and its duration, blood culture results, duration of indwelling central lines and mechanical ventilation, and mortality before discharge. Small for GA (SGA) was defined as BW under the 10^th^ percentile using Norwegian growth charts.^25^ We defined severe BPD as receiving any respiratory support, other than supplemental oxygen only, at 36 weeks post-menstrual age (PMA) and severe retinopathy of prematurity (ROP) as treated with laser or anti-vascular endothelial growth factor in either eye.

We extracted data on all antibiotics administered, and divided them into five different groups. Narrow-spectrum β-lactams included benzylpenicillin, ampicillin and cloxacillin. Broad-spectrum β-lactams included third-generation cephalosporins and carbapenems; second-generation cephalosporins and piperacillin/tazobactam were not used for very preterm infants in Norway during the study period.^22^ The final three antibiotic groups were aminoglycosides (gentamicin or tobramycin), first-generation cephalosporins (cefalotin or cefazolin) and vancomycin, which is the only glycopeptide licensed in Norway.

Antibiotic exposure within each health region is presented as days of treatment (DOT) per 1000 patient days (PD).^26^ We present data on DOT for any antibiotic therapy per 1000 PD, and data on DOT for specific antibiotic groups per 1000 PD. Since a combination of different antibiotic groups is often used, the cumulative days of DOT for the specific antibiotic groups per 1000 PD will exceed the DOT for any antibiotic per 1000 PD.

We similarly present the burden of invasive therapies as days of mechanical ventilation per 1000 PDs and days of indwelling central lines per 1000 PDs. Both these variables are associated with increased risk of infection and antibiotic use.^27,28^

Since antibiotic consumption also depends on infection rates, we present regional rates of sepsis in the study population. Culture-positive sepsis was defined as growth of a bacterium or fungus that is commonly considered pathogenic in blood culture and antibiotic treatment for at least 5 days or death before 5 days during this episode in an infant with clinical signs of sepsis.^23,29^ A culture-positive sepsis with onset of therapy within the first 3 days of life was defined as early-onset sepsis (EOS) and after 3 days of life as late-onset sepsis (LOS).^29,30^ For the definition of LOS with CoNS, we also required a C-reactive protein (CRP) level of >10 mg/L to distinguish from contamination.^29^ In the case of EOS, CoNS were always classified as contaminants.^31^ Sepsis-attributable death was defined as death within 7 days of a positive blood culture.^32^

### Statistical methods

Statistical analyses were performed with SPSS 29.0 (SPSS, Armonk, NY, USA). Results are presented as medians with IQRs or proportions (%). Differences between groups were analysed with non-parametric (Kruskal–Wallis or Mann–Whitney *U*-test) or parametric (ANOVA and *t*-test) tests for continuous variables, and Fisher’s exact or chi-squared tests for categorical data, as appropriate. Trends in antibiotics use in each health region were analysed with linear logistic regression and adjusted for CRIB2 score as a relevant confounder. Two-tailed *P* values of <0.05 were considered statistically significant.

## Results

We included 5296 infants: 3646 (69%) born at 28–31 weeks and 1650 (31%) born before 28 weeks gestation. The median GA and BW were 29 weeks and 1.2 kg, respectively. Infants shared similar baseline characteristics across all four health regions, including illness severity scores (CRIB2), sex distribution and proportion of SGA infants. There were some differences in rates of Caesarean deliveries and antenatal steroid use (Table 1).

**Table 1.** Background characteristics of 5296 preterm infants born before 32 weeks gestation by four Norwegian health regions

	South-East *n* = 2919	West *n* = 1217	Mid *n* = 730	North *n* = 430	*P* value
Background					
* *GA (weeks), median (IQR)	29 + 3 (27 + 1–31 + 0)	29 + 3 (27 + 3–30 + 6)	29 + 4 (27 + 3–31 + 0)	29 + 4 (27 + 2–31 + 4)	0.963
* *BW (g), median (IQR)	1199 (875–1505)	1225 (903–1516)	1225 (899–1510)	1200 (895–1730)	0.447
* *SGA (%)	27.2	24.9	25.2	29.1	0.520
* *Apgar 5 min, median (IQR)	8 (7–9)	8 (6–9)	8 (7–9)	8 (7–9)	0.272
* *CRIB 2 score, median (IQR)	7 (4–10)	6 (4–9)	6 (3–9)	6 (4–10)	0.234
* *Caesarean delivery (%)	41.9	60.1	71.8	45.6	<0.001^a^
* *Antenatal steroids (%)	85.2	86.2	87.9	81.4	0.021^b^
* *Female (%)	44.2	46.1	44.4	47.9	0.402
Exposures					
* *Days of mechanical ventilation per 1000 PD	113	62	136	92	0.104
* *Days of central lines per 1000 PD	273	100	182	149	<0.001^c^
* *Days of hospitalization, median (IQR)	49 (33–74)	50 (34–73)	50 (34–75)	56 (41–79)	<0.001^d^
Outcomes					
* *Severe BPD (%)	13.9	9.8	14.1	17.7	<0.001^c^
* *Severe ROP (%)	2.4	4.3	2.2	3.0	0.006^c^
* *Surgically treated NEC (%)	1.7	1.8	1.4	2.3	0.646
* *In-hospital mortality (%)	8.5	9.0	9.5	8.4	0.828
* *Age at death (days), median (IQR)	7 (2–21)	6 (1–16)	3 (1–20)	4 (2–20)	0.162

Severe BPD: need for any respiratory support, other than O_2_ only, at 36 weeks + 0–6 days PMA; one value was missing. Severe ROP (receiving therapy with laser or anti-vascular endothelial growth factor): nine values were missing. Central lines (include any of the following: umbilical artery and/or vein, peripherally inserted central catheter and central venous catheter): four values were missing. CRIB 2: 290 values were missing

^a^Significant differences between all regions except between South-East and North.

^b^Significant differences between Mid and North.

^c^Significant differences between West and all other regions.

^d^Significant differences between North and all other regions.

**Figure 1.**
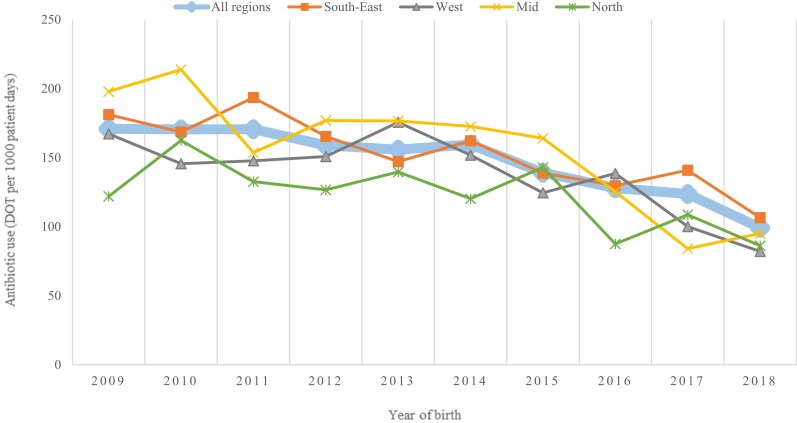
Trends in antibiotic consumption among very preterm infants in four Norwegian health regions, 2009–18. This figure appears in colour in the online version of *JAC* and in black and white in the print version of *JAC*.

**Figure 2.**
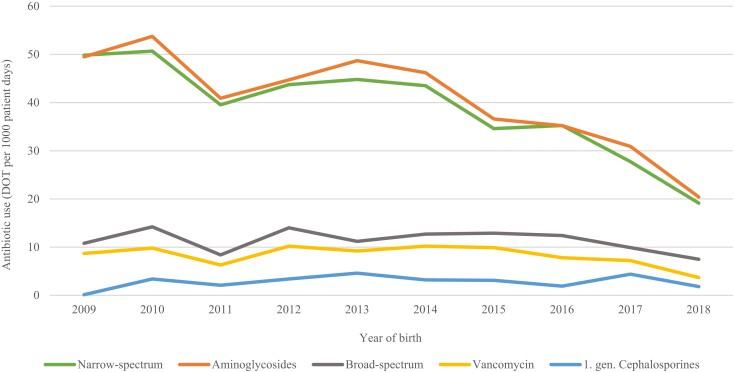
Trends in consumption of different antibiotic groups among very preterm infants in Norway, 2009–18. Narrow-spectrum β-lactams: benzylpenicillin, ampicillin and cloxacillin, Broad-spectrum β-lactams: third-generation cephalosporins and carbapenems. Aminoglycosides (gentamicin or tobramycin). This figure appears in colour in the online version of *JAC* and in black and white in the print version of *JAC*.

Around 80% of very preterm infants received antibiotic therapy during their stay in the NICU and the median treatment duration was 7 days (IQR 2–11). Among infants born before 28 weeks gestation, 98% received antibiotics in comparison with 72% in those born between 28 and 31 weeks (*P* < 0.001) (Table S1, available as Supplementary data at *JAC* Online). The overall trends in antibiotic consumption across all four regions are displayed in Figure 1. The proportion of infants receiving antibiotics decreased from 84% in 2009 to 70% in 2018 (*P* < 0.001) and the proportion of infants receiving antibiotic therapy for more than 5 days decreased from 48% in 2009 to 18% in 2018 (*P* < 0.001). Figure 2 displays trends in consumption of the different antibiotic groups. The most frequently used antibiotics were narrow-spectrum β-lactams and aminoglycosides, and the overall reduction in antibiotic consumption during the study period was predominantly due to a reduction in consumption of these two groups (*P* < 0.001 for both). In contrast, the consumption of broad-spectrum β-lactams (*P* = 0.308) and vancomycin (*P* = 0.292) remained stable throughout the study period.

There were considerable regional differences in the total antibiotic consumption (Table 2), the South-East and Mid regions having the highest overall consumption, with 159 and 161 DOT per 1000 PD, respectively. There were also marked variations in the consumption of broad-spectrum β-lactams (South-East highest), first-generation cephalosporins (North highest) and vancomycin (South-East highest).

**Table 2.** Antibiotic consumption and regional variations, expressed as DOT per 1000 PD, among very preterm infants born before 32 weeks gestation in Norway

DOT per 1000 PD	Norway	South-East	West	Mid	North	*P* value
All antibiotic therapy^a^	149	159	141	161	122	0.012^b^
Narrow-spectrum β-lactams	108	103	109	108	70	<0.001^c^
Broad-spectrum β-lactams	34	42	24	31	17	<0.001^d^
Aminoglycosides	102	100	108	108	91	0.038^e^
First-generation cephalosporins	3	1	1	1	25	<0.001^c^
Vancomycin	27	36	18	22	8	<0.001^c^

Narrow-spectrum β-lactams: benzylpenicillin, ampicillin and cloxacillin. Broad-spectrum β-lactams: third-generation cephalosporins (∼85%) and carbapenems (∼15%).

^a^When calculating ‘all antibiotics’, 1 day with combination therapy is counted as 1 day.

^b^Significant differences between North and Mid, between North and South-East, and between West and South-East.

^c^Significant differences between North and all other regions.

^d^Significant differences between South-East and all other regions.

^e^Significant differences between North and South-East, and between North and West.

Table 1 presents potential risk factors for neonatal sepsis and Table 3 presents rates of the different types of sepsis and their sepsis-attributable mortality. There were significant regional differences in exposure to central lines, the lowest rate being in the West region. This region also had lower rates of CoNS-LOS compared with the three other regions. The consumption of vancomycin and first-generation cephalosporins varied significantly between the North region and the other three regions, but CoNS-LOS-attributable mortality rates were similar (Tables 2 and 3). Finally, rates of Gram-negative LOS and their sepsis-attributable mortality were similar between regions, but the use of broad-spectrum β-lactam antibiotics varied markedly, from 42 DOT/1000 PD in the South-East region to 17 DOT/1000 PD in the North region.

**Table 3.** Rates of sepsis and sepsis-attributable mortality across four Norwegian health regions

	South-East *n* = 2919	West *n* = 1217	Mid *n* = 730	North *n* = 430	*P* value
All culture-positive EOS, *n* (%)^a^	38 (1.3)	18 (1.5)	20 (2.7)	8 (1.9)	0.076
* *Sepsis-attributable mortality, *n* (%)	12/38 (31.6)	3/18 (16.7)	2/20 (10)	3/8 (37.5)	0.201
All culture-positive LOS, *n* (%)^a^	340 (11.6)	67 (5.5)	36 (4.9)	50 (11.6)	<0.001^b^
* *Sepsis-attributable mortality, *n* (%)	31/340 (9.1)	9/67 (13.4)	8/36 (22.2)	6/50 (12)	0.650
Gram-negative LOS, *n* (%)^c^	62 (18.2)	16 (23.9)	10 (27.8)	7 (14.0)	0.240
* *Sepsis-attributable mortality, *n* (%)	9/62 (14.5)	2/16 (12.5)	2/10 (20.0)	2/7 (28.6)	0.756
Gram-positive, excluding CoNS- LOS, *n* (%)^c^	106 (31.2)	31 (46.3)	11 (30.6)	15 (30.0)	0.015^d^
* *Sepsis-attributable mortality, *n* (%)	5/106 (4.7)	3/31 (3.2)	1/11 (9.1)	1/15 (6.7)	0.872
CoNS LOS, *n* (%)^c^	165 (48.5)	20 (29.9)	14 (38.9)	25 (50.0)	<0.001^b^
* *Sepsis-attributable mortality, *n* (%)	5/165 (3.0)	2/20 (10.0)	1/14 (7.1)	1/25 (4.0)	0.175

^a^Percentage of the study population.

^b^Significant differences between South-East and West, between South-East and Mid, between West and North, and between Mid and North.

^c^Percentage of all culture-positive LOS cases.

^d^Significant differences between South-East and West.

## Discussion

In this unselected population of all very preterm infants in Norway, the overall consumption of antibiotics decreased markedly during the 10 year period, but the consumption of broad-spectrum β-lactams, first-generation cephalosporins and vancomycin remained stable. However, there was a considerable variation between health regions, both in the overall antibiotic consumption as well as in consumption of different antibiotic groups. Despite these variations, sepsis-attributable death and the overall in-hospital mortality were similar in all regions.

Measurement of antibiotic consumption in DOT, if normalized to PD, is the preferred metric of antibiotic use in children and is often used for benchmarking between institutions or regions. An overall antibiotic consumption of 149 DOT/1000 PD among very preterm infants in our study is at the lower end compared with studies with similar populations.^19,30,33–36^ However, caution should be exercised when making comparisons between institutions or other regions with different case mixes.^34^ In our study, background data did not indicate obvious signs of case mix and we used population-based data for all regions. Still, we note that neonatal surgery is centralized to two hospitals located in South-East and Mid Norway. Transfer of preterm infants in need of surgery, e.g. for necrotizing enterocolitis, may partly explain the higher overall antibiotic consumption in these regions. Moreover, the median duration of stay for infants was longer in the North, potentially having an impact on DOT per 1000 PD by increasing the denominator.

Narrow-spectrum β-lactam antibiotics and aminoglycosides were the most commonly used antibiotics across all regions. The combination of these two antibiotic groups has long been standard empirical therapy for EOS in Norway^37^ and in many other countries.^38,39^ However, consumption of these antibiotics declined markedly during the study period, in particular in terms of prolonged empirical therapy. We believe this reflects the increased attention to the potential adverse clinical outcomes of prolonged early empirical antibiotics first reported by Cotten and co-workers in 2009^7^ and subsequently reported by others,^6,16^ including recent data from this Norwegian cohort of very preterm infants.^17^ Quality improvement projects in Norway focusing on antibiotic overuse in infants that took place during the study period may also have contributed to a more cautious use of antibiotics in the preterm population.^40,41^ Similar findings have been reported by single-centre studies in Sweden and the Netherlands, showing a marked decrease in antibiotic consumption following antibiotic stewardship efforts in the hospitals.^35,42^

For LOS, in comparison with EOS, a much larger heterogenicity in empirical treatment regimens has been reported.^8,43,44^ In our study, we did not differentiate between empirical therapy and targeted antibiotic use for blood culture-proven sepsis, but it is well known that empirical therapy constitutes the largest proportion of antibiotic use. There were clear regional variations in the choice of antibiotics after the first week of life, in particular for vancomycin, broad-spectrum β-lactams and first-generation cephalosporins. National blood culture data from Norway report that all Group B streptococci are susceptible to penicillin, the vast majority of *Staphylococcus aureus* isolates are susceptible to oxacillin and around 95% of all *Escherichia coli* are susceptible to gentamicin.^40,45^ Antibiotic susceptibility data specifically from neonatal blood cultures are not available from all Norwegian regions. However, data from one large NICU reported a lower gentamicin susceptibility rate for *E. coli* than in older children and adults.^46^ Previous Norwegian reports have shown that the majority (>80%) of hospital CoNS isolates from infants are resistant to oxacillin *in vitro*, but almost uniformly susceptible to vancomycin.^46–48^ The crude numbers of sepsis-attributable mortality in this study were small and our study was not powered for this outcome, but we found no regional differences, neither overall nor for specific pathogens. Thus, based on both national susceptibility and outcome data, the variations observed in our study predominantly seem to reflect variations in antibiotic policies rather than substantial differences in care complexity and susceptibility patterns of invasive pathogens. A variation in use of broad-spectrum antibiotics, without obvious large differences in case mixes, is also reported from other studies.^6,26^ Recently, a large US study reported a 4-fold variation in DOT for antibiotics per 1000 PD across NICUs with similar care complexity.^34^ A potential overuse of third-generation cephalosporins or carbapenems increases selective pressure for multi-resistant Gram-negative organisms, and is associated with increased risk for invasive candidiasis.^8,49^ Reports also indicate that broad-spectrum β-lactams often do not offer a benefit in antimicrobial spectrum over a narrow-spectrum β-lactam and an aminoglycoside in combination,^18^ and some studies even indicate increased morbidity or mortality.^50^

CoNS is the most prevalent pathogen causing LOS among very preterm infants in Norway,^29^ as in many other countries.^42,51^ CoNS-LOS has been associated with a later increased risk of severe BPD,^29^ but this association is not consistently reported.^52,53^ However, these commensal bacteria rarely cause fulminant sepsis, but vancomycin is frequently used as empirical therapy.^1,8,26^ Indiscriminate use of vancomycin has been linked to the emergence of vancomycin-resistant organisms.^54^ Some studies also report that vancomycin has a higher nephrotoxic and ototoxic potential in preterm infants compared with β-lactams,^55–57^ whereas others do not report increased risk of toxicity.^58,59^ The empirical use of vancomycin is therefore controversial. Several large multicentre studies have failed to demonstrate a survival benefit with empirical vancomycin therapy for CoNS bloodstream infections versus alternatives with β-lactam antibiotics combined with an aminoglycoside or versus delayed vancomycin therapy.^49,60^ In line with this, we found a similar low CoNS-LOS attributable mortality across all regions despite marked variations in vancomycin consumption. Studies from the Netherlands report that the majority of oxacillin-resistant CoNS sepsis cases can successfully be treated with first-generation cephalosporins, antibiotics that also provide Gram-negative coverage.^61–63^ This strategy was chosen in the North region and was therefore associated with markedly higher use of first-generation cephalosporins compared with the other regions. Choice of an adequate empirical regimen for LOS needs to balance the higher risk of sepsis-attributable mortality with Gram-negative bacteria, risk of toxicity and antibiotic resistance development with different regimens. The American Academy of Pediatrics and Canadian guidelines for neonatal sepsis discourage vancomycin as empirical LOS therapy if the prevalence of MRSA in the community is low.^10,64^

The primary strength of this study is prospective collection of data on a daily basis with standardized online registration. As data on antibiotic therapy were registered on a daily basis, consistent underestimation of treatment is unlikely. The 10 year study period allows evaluation of trends, and the population-based design avoids selection bias.

Inaccuracy of registered data together with a large number of physicians involved represents the main limitation. Moreover, we did not have antibiotic susceptibility profiles of isolates causing sepsis. Finally, we did not have detailed data of the proportion of empirical therapy versus therapy for proven infections.

### Conclusions

This study demonstrates a marked decline in overall antibiotic consumption during the study period. Still, there were substantial regional variations in antibiotic consumption among very preterm infants in Norway, despite similar rates of sepsis and sepsis-attributable mortality, highlighting the need for coordinated ASP strategies. There is still a low prevalence of multi-resistant organisms in Norwegian NICUs, offering a time-critical opportunity to preserve a favourable ecological situation. Standardization of national treatment guidelines combined with adequate ASPs are needed to safely minimize broad-spectrum antimicrobial exposure and limit the development of antimicrobial resistance.

## Supplementary Material

dkad358_Supplementary_Data

## Supplementary data

Table S1 is available as Supplementary data at *JAC* Online.

## References

[1] Mukhopadhyay S , Sengupta S, Puopolo KM. Challenges and opportunities for antibiotic stewardship among preterm infants. Arch Dis Child Fetal Neonatal Ed 2019; 104: F327–32. 10.1136/archdischild-2018-315412 30425110 PMC6491257

[2] Dierikx TH , Deianova N, Groen J et al Association between duration of early empiric antibiotics and necrotizing enterocolitis and late-onset sepsis in preterm infants: a multicenter cohort study. Eur J Pediatr 2022; 181: 3715–24. 10.1007/s00431-022-04579-5 35927379 PMC9508214

[3] Giannoni E , Dimopoulou V, Klingenberg C et al Analysis of antibiotic exposure and early-onset neonatal sepsis in Europe, North America, and Australia. JAMA Netw Open 2022; 5: e2243691. 10.1001/jamanetworkopen.2022.43691 36416819 PMC9685486

[4] Schulman J , Dimand RJ, Lee HC et al Neonatal intensive care unit antibiotic use. Pediatrics 2015; 135: 826–33. 10.1542/peds.2014-3409 25896845

[5] Flannery DD , Ross RK, Mukhopadhyay S et al Temporal trends and center variation in early antibiotic use among premature infants. JAMA Netw Open 2018; 1: e180164. 10.1001/jamanetworkopen.2018.0164 30646054 PMC6324528

[6] Esaiassen E , Fjalstad JW, Juvet LK et al Antibiotic exposure in neonates and early adverse outcomes: a systematic review and meta-analysis. J Antimicrob Chemother 2017; 72: 1858–70. 10.1093/jac/dkx088 28369594

[7] Cotten CM , Taylor S, Stoll B et al Prolonged duration of initial empirical antibiotic treatment is associated with increased rates of necrotizing enterocolitis and death for extremely low birth weight infants. Pediatrics 2009; 123: 58–66. 10.1542/peds.2007-3423 19117861 PMC2760222

[8] Litz JE , Goedicke-Fritz S, Härtel C et al Management of early- and late-onset sepsis: results from a survey in 80 German NICUs. Infection 2019; 47: 557–64. 10.1007/s15010-018-1263-9 30607897

[9] Schulman J , Profit J, Lee HC et al Variations in neonatal antibiotic use. Pediatrics 2018; 142: e20180115. 10.1542/peds.2018-0115 30177514 PMC6188671

[10] Ting JY , Autmizguine J, Dunn MS et al Practice summary of antimicrobial therapy for commonly encountered conditions in the neonatal intensive care unit: a Canadian perspective. Front Pediatr 2022; 10: 894005. 10.3389/fped.2022.894005 35874568 PMC9304938

[11] NordForsk . ALLTogether—a European treatment protocol for children and young adults with acute lymphoblastic leukaemia (ALL). https://www.nordforsk.org/projects/alltogether-european-treatment-protocol-children-and-young-adults-acute-lymphoblastic.

[12] Korang SK , Safi S, Nava C et al Antibiotic regimens for early-onset neonatal sepsis. Cochrane Database Syst Rev 2021; issue 5: CD013837. 10.1002/14651858.CD013837.pub2 33998666 PMC8127574

[13] Korang SK , Safi S, Nava C et al Antibiotic regimens for late-onset neonatal sepsis. Cochrane Database Syst Rev 2021; issue 5: CD013836. 10.1002/14651858.CD013836.pub2 33998665 PMC8127057

[14] Fjalstad JW , Esaiassen E, Juvet LK et al Antibiotic therapy in neonates and impact on gut microbiota and antibiotic resistance development: a systematic review. J Antimicrob Chemother 2018; 73: 569–80. 10.1093/jac/dkx426 29182785

[15] Ting JY , Roberts A, Sherlock R et al Duration of initial empirical antibiotic therapy and outcomes in very low birth weight infants. Pediatrics 2019; 143: e20182286. 10.1542/peds.2018-2286 30819968

[16] Kuppala VS , Meinzen-Derr J, Morrow AL et al Prolonged initial empirical antibiotic treatment is associated with adverse outcomes in premature infants. J Pediatr 2011; 159: 720–5. 10.1016/j.jpeds.2011.05.033 21784435 PMC3193552

[17] Vatne A , Hapnes N, Stensvold HJ et al Early empirical antibiotics and adverse clinical outcomes in infants born very preterm: a population-based cohort. J Pediatr 2022; 253: 107–14. 10.1016/j.jpeds.2022.09.029 36179887

[18] Carr JP , Burgner DP, Hardikar RS et al Empiric antibiotic regimens for neonatal sepsis in Australian and New Zealand neonatal intensive care units. J Paediatr Child Health 2017; 53: 680–4. 10.1111/jpc.13540 28421643

[19] Hamdy RF , Bhattarai S, Basu SK et al Reducing vancomycin use in a level IV NICU. Pediatrics 2020; 146: e20192963. 10.1542/peds.2019-2963 32611807

[20] de With K , Allerberger F, Amann S et al Strategies to enhance rational use of antibiotics in hospital: a guideline by the German Society for Infectious Diseases. Infection 2016; 44: 395–439. 10.1007/s15010-016-0885-z 27066980 PMC4889644

[21] Stocker M , Klingenberg C, Navér L et al Less is more: antibiotics at the beginning of life. Nat Commun 2023; 14: 2423. 10.1038/s41467-023-38156-7 37105958 PMC10134707

[22] Norwegian Neonatal Network . Norwegian Neonatal Medicine Quality Registry. https://www.kvalitetsregistre.no/register/skade-og-intensiv-barn/norsk-nyfodtmedisinsk-kvalitetsregister.

[23] Stensvold HJ , Klingenberg C, Stoen R et al Neonatal morbidity and 1-year survival of extremely preterm infants. Pediatrics 2017; 139: e20161821. 10.1542/peds.2016-1821 28228499

[24] Parry G , Tucker J, Tarnow-Mordi W. CRIB II: an update of the clinical risk index for babies score. Lancet 2003; 361: 1789–91. 10.1016/S0140-6736(03)13397-1 12781540

[25] Skjaerven R , Gjessing HK, Bakketeig LS. Birthweight by gestational age in Norway. Acta Obstet Gynecol Scand 2000; 79: 440–9. 10.1034/j.1600-0412.2000.079006440.x 10857867

[26] Kramer TS , Salm F, Schwab F et al Reduction of antibacterial use in patients with very low birth weight on German NICUs after implementation of a mandatory surveillance system. A longitudinal study with national data from 2013 to 2019. J Infect 2022; 85: 8–16. 10.1016/j.jinf.2022.05.009 35580752

[27] Dong Y , Speer CP. Late-onset neonatal sepsis: recent developments. Arch Dis Child Fetal Neonatal Ed 2015; 100: F257–63. 10.1136/archdischild-2014-306213 25425653 PMC4413803

[28] El Manouni El Hassani S , Berkhout DJC, Niemarkt HJ et al Risk factors for late-onset sepsis in preterm infants: a multicenter case-control study. Neonatology 2019; 116: 42–51. 10.1159/000497781 30947195 PMC6690411

[29] Huncikova Z , Vatne A, Stensvold HJ et al Late-onset sepsis in very preterm infants in Norway in 2009–2018: a population-based study. Arch Dis Child Fetal Neonatal Ed 2023; 108: 478–84. 10.1136/archdischild-2022-324977 36732047 PMC10447404

[30] Berardi A , Zinani I, Rossi C et al Antibiotic use in very low birth weight neonates after an antimicrobial stewardship program. Antibiotics (Basel) 2021; 10: 411. 10.3390/antibiotics10040411 33918796 PMC8070476

[31] Stoll BJ , Hansen NI, Sánchez PJ et al Early onset neonatal sepsis: the burden of group B streptococcal and *E. coli* disease continues. Pediatrics 2011; 127: 817–26. 10.1542/peds.2010-2217 21518717 PMC3081183

[32] Levit O , Bhandari V, Li FY et al Clinical and laboratory factors that predict death in very low birth weight infants presenting with late-onset sepsis. Pediatr Infect Dis J 2014; 33: 143–6. 10.1097/INF.0000000000000024 24418836 PMC3917323

[33] Cantey JB , Wozniak PS, Pruszynski JE et al Reducing unnecessary antibiotic use in the neonatal intensive care unit (SCOUT): a prospective interrupted time-series study. Lancet Infect Dis 2016; 16: 1178–84. 10.1016/S1473-3099(16)30205-5 27452782

[34] Singh P , Steurer MA, Cantey JB et al Hospital-level antibiotic use and complexity of care among neonates. J Pediatric Infect Dis Soc 2020; 9: 656–63. 10.1093/jpids/piz091 31879765 PMC7974016

[35] Gustavsson L , Lindquist S, Elfvin A et al Reduced antibiotic use in extremely preterm infants with an antimicrobial stewardship intervention. BMJ Paediatr Open 2020; 4: e000872. 10.1136/bmjpo-2020-000872 PMC7722820 33324764

[36] Ting JY , Paquette V, Ng K et al Reduction of inappropriate antimicrobial prescriptions in a tertiary neonatal intensive care unit after antimicrobial stewardship care bundle implementation. Pediatr Infect Dis J 2019; 38: 54–9. 10.1097/INF.0000000000002039 30531528

[37] Fjalstad JW , Stensvold HJ, Bergseng H et al Early-onset sepsis and antibiotic exposure in term infants: a nationwide population-based study in Norway. Pediatr Infect Dis J 2016; 35: 1–6. 10.1097/INF.0000000000000906 26368059

[38] Puopolo KM , Benitz WE, Zaoutis TE et al Management of neonates born at ≥34 6/7 weeks’ gestation with suspected or proven early-onset bacterial sepsis. Pediatrics 2018; 142: 12. 10.1542/peds.2018-2894 30455342

[39] NICE . Neonatal infection: antibiotics for prevention and treatment. NICE guideline [NG195]. 2021. https://www.nice.org.uk/guidance/ng195.

[40] Dretvik T , Solevåg AL, Finvåg A et al Active antibiotic discontinuation in suspected but not confirmed early-onset neonatal sepsis—a quality improvement initiative. Acta Paediatr 2020; 109: 1125–30. 10.1111/apa.15202 31999863

[41] Vatne A , Klingenberg C, Øymar K et al Reduced antibiotic exposure by serial physical examinations in term neonates at risk of early-onset sepsis. Pediatr Infect Dis J 2020; 39: 438–43. 10.1097/INF.0000000000002590 32301920

[42] Jansen SJ , van der Hoeven A, van den Akker T et al A longitudinal analysis of nosocomial bloodstream infections among preterm neonates. Eur J Clin Microbiol Infect Dis 2022; 41: 1327–36. 10.1007/s10096-022-04502-8 36178568 PMC9556429

[43] Leroux S , Zhao W, Bétrémieux P et al Therapeutic guidelines for prescribing antibiotics in neonates should be evidence-based: a French national survey. Arch Dis Child 2015; 100: 394–8. 10.1136/archdischild-2014-306873 25628457

[44] Lutsar I , Chazallon C, Carducci FI et al Current management of late onset neonatal bacterial sepsis in five European countries. Eur J Pediatr 2014; 173: 997–1004. 10.1007/s00431-014-2279-5 24522326

[45] NORM/NORM-VET . NORM/NORM-VET 2021: Usage of Antimicrobial Agents and Occurrence of Antimicrobial Resistance in Norway. 2022.https://www.fhi.no/en/publ/2022/norm-og-norm-vet-usage-of-antimicrobial-agents-and-occurrence-of-antimicrob/.

[46] Størdal EH , Solevåg AL, Bjørnholt JV et al Sepsis treatment options identified by 10-year study of microbial isolates and antibiotic susceptibility in a level-four neonatal intensive care unit. Acta Paediatr 2022; 111: 519–26. 10.1111/apa.16189 34787905

[47] Klingenberg C , Aarag E, Rønnestad A et al Coagulase-negative staphylococcal sepsis in neonates. Association between antibiotic resistance, biofilm formation and the host inflammatory response. Pediatr Infect Dis J 2005; 24: 817–22. 10.1097/01.inf.0000176735.20008.cd 16148849

[48] Klingenberg C , Rønnestad A, Anderson AS et al Persistent strains of coagulase-negative staphylococci in a neonatal intensive care unit: virulence factors and invasiveness. Clin Microbiol Infect 2007; 13: 1100–11. 10.1111/j.1469-0691.2007.01818.x 17850346

[49] Ericson JE , Thaden J, Cross HR et al No survival benefit with empirical vancomycin therapy for coagulase-negative staphylococcal bloodstream infections in infants. Pediatr Infect Dis J 2015; 34: 371–5. 10.1097/INF.0000000000000573 25760564 PMC4357312

[50] Clark RH , Bloom BT, Spitzer AR et al Empiric use of ampicillin and cefotaxime, compared with ampicillin and gentamicin, for neonates at risk for sepsis is associated with an increased risk of neonatal death. Pediatrics 2006; 117: 67–74. 10.1542/peds.2005-0179 16396862

[51] Giannoni E , Agyeman PKA, Stocker M et al Neonatal sepsis of early onset, and hospital-acquired and community-acquired late onset: a prospective population-based cohort study. J Pediatr 2018; 201: 106–14. 10.1016/j.jpeds.2018.05.048 30054165

[52] Cantey JB , Anderson KR, Kalagiri RR et al Morbidity and mortality of coagulase-negative staphylococcal sepsis in very-low-birth-weight infants. World J Pediatr 2018; 14: 269–73. 10.1007/s12519-018-0145-7 29536341

[53] Ohlin A , Björkman L, Serenius F et al Sepsis as a risk factor for neonatal morbidity in extremely preterm infants. Acta Paediatr 2015; 104: 1070–6. 10.1111/apa.13104 26118325

[54] Lawrence SL , Roth V, Slinger R et al Cloxacillin versus vancomycin for presumed late-onset sepsis in the neonatal intensive care unit and the impact upon outcome of coagulase negative staphylococcal bacteremia: a retrospective cohort study. BMC Pediatr 2005; 5: 49. 10.1186/1471-2431-5-49 16375769 PMC1343548

[55] Dawoud TH , Khan N, Afzal U et al Assessment of initial vancomycin trough levels and risk factors of vancomycin-induced nephrotoxicity in neonates. Eur J Hosp Pharm 2022; 29: 44–9. 10.1136/ejhpharm-2019-002181 34930794 PMC8717783

[56] Marissen J , Fortmann I, Humberg A et al Vancomycin-induced ototoxicity in very-low-birthweight infants. J Antimicrob Chemother 2020; 75: 2291–8. 10.1093/jac/dkaa156 32464660

[57] Vella-Brincat JW , Begg EJ, Robertshawe BJ et al Are gentamicin and/or vancomycin associated with ototoxicity in the neonate? A retrospective audit. Neonatology 2011; 100: 186–93. 10.1159/000324857 21455009

[58] Lestner JM , Hill LF, Heath PT et al Vancomycin toxicity in neonates: a review of the evidence. Curr Opin Infect Dis 2016; 29: 237–47. 10.1097/QCO.0000000000000263 26895572

[59] Constance JE , Balch AH, Stockmann C et al A propensity-matched cohort study of vancomycin-associated nephrotoxicity in neonates. Arch Dis Child Fetal Neonatal Ed 2016; 101: F236–43. 10.1136/archdischild-2015-308459 26400103

[60] Isaacs D . A ten year, multicentre study of coagulase negative staphylococcal infections in Australasian neonatal units. Arch Dis Child Fetal Neonatal Ed 2003; 88: F89–93. 10.1136/fn.88.2.F89 12598493 PMC1721527

[61] Hemels MA , van den Hoogen A, Verboon-Maciolek MA et al A seven-year survey of management of coagulase-negative staphylococcal sepsis in the neonatal intensive care unit: vancomycin may not be necessary as empiric therapy. Neonatology 2011; 100: 180–5. 10.1159/000324852 21455008

[62] Fleer A , Hemels MA, Paauw A et al Reduced expression of PBP-2A by neonatal *mecA*-positive coagulase-negative staphylococci (CoNS) blood isolates: β-lactams are useful first-line agents for the treatment of neonatal CoNS sepsis, restricting the use of vancomycin. J Antimicrob Chemother 2012; 67: 1616–8. 10.1093/jac/dks092 22438436

[63] Krediet TG , Jones ME, Gerards LJ et al Clinical outcome of cephalothin versus vancomycin therapy in the treatment of coagulase-negative staphylococcal septicemia in neonates: relation to methicillin resistance and *mec A* gene carriage of blood isolates. Pediatrics 1999; 103: E29. 10.1542/peds.103.3.e29 10049985

[64] American Academy of Pediatrics—Committee on Infectious Diseases, the Pediatric Infectious Diseases Society . Choosing Wisely. https://www.choosingwisely.org/societies/american-academy-of-pediatrics-committee-on-infectious-diseases-and-the-pediatric-infectious-diseases-society.

