# Supplementary material for: Variation in antibiotic consumption in very preterm infants—a 10 year population-based study
Source: J Antimicrob Chemother. 2023 Nov 21;79(1):143–50. doi: 10.1093/jac/dkad358 (PMC10761275; doi:10.1093/jac/dkad358)
Supplement: dkad358_Supplementary_Data [file dkad358_supplementary_data.docx]

**Supplementary Table 1.** Antibiotic consumption among Norwegian very preterm infants, presented for each gestational week, for the period 2009-2018

| Gestational age (weeks) | DOT all antibiotics per 1000 patient days | Proportion (%) receiving any antibiotics | Days of all antibiotic treatment, median (IQR) |
| --- | --- | --- | --- |
| 23 (n=168) | 280 | 98 | 12 (4-29) |
| 24 (n=272) | 255 | 98 | 18 (9-30) |
| 25 (n=286) | 218 | 99 | 17 (9-27) |
| 26 (n=429) | 182 | 98 | 12 (7-19) |
| 27 (n=481) | 158 | 98 | 9 (5-15) |
| 28 (n=597) | 136 | 94 | 6 (4-10) |
| 29 (n=770) | 113 | 85 | 5 (3-8) |
| 30 (n=961) | 100 | 72 | 3 (0-6) |
| 31 (n=1318) | 77 | 53 | 2 (0-4) |

There were only 14 infants cared for at GA 22 weeks, of which nine died within eight days
